# From Suspicion to Confirmation: An Original Study on a Complete Diagnostic Pathway for Ectopic Pregnancy

**DOI:** 10.3390/jcm15020507

**Published:** 2026-01-08

**Authors:** Marian Valentin Zorilă, Dominic-Gabriel Iliescu, George-Lucian Zorilă, Daniel Pirici, Anca-Maria Istrate-Ofiţeru, Camelia-Gabriela Roşu, Cristina Jana Busuioc, Laurențiu Mogoantă, Vanda Roxana Nimigean, Răzvan Grigoraș Căpitănescu, Elena Iuliana Anamaria Berbecaru, Roxana-Cristina Drăgușin, Maria-Cristina Comănescu, Stefan Paitici

**Affiliations:** 1 Department of Forensic Medicine, University of Medicine and Pharmacy of Craiova, 200349 Craiova, Romania; valentin.zorila@umfcv.ro; 2 Department of Obstetrics and Gynecology, University of Medicine and Pharmacy of Craiova, 200349 Craiova, Romania; dominic.iliescu@umfcv.ro (D.-G.I.); lucian.zorila@umfcv.ro (G.-L.Z.); roxana.dragusin@umfcv.ro (R.-C.D.); 3 Department of Histology, Faculty of Medicine, University of Medicine and Pharmacy of Craiova, 200349 Craiova, Romania; daniel.pirici@umfcv.ro (D.P.); camelia.rosu@umfcv.ro (C.-G.R.); cristina.busuioc@umfcv.ro (C.J.B.); laurentiu.mogoanta@umfcv.ro (L.M.); 4 Research Center for Microscopic Morphology and Immunology, University of Medicine and Pharmacy of Craiova, 200349 Craiova, Romania; 5 Department of Oral Rehabilitation, Faculty of Dentistry, Carol Davila University of Medicine and Pharmacy, 050474 Bucharest, Romania; vanda.nimigean@umfcd.ro; 6 Doctoral School, University of Medicine and Pharmacy of Craiova, 200349 Craiova, Romania; elena.berbecaru@umfcv.ro; 7 Department of Anatomy, University of Medicine and Pharmacy of Craiova, 200349 Craiova, Romania; maria.comanescu@umfcv.ro; 8 Department of Surgery, University of Medicine and Pharmacy of Craiova, 200349 Craiova, Romania; stefan.paitici@umfcv.ro

**Keywords:** ectopic pregnancy, ultrasonography, methotrexate, surgical management, histopathological examination

## Abstract

**Background/Objectives:** Ectopic pregnancy (EP) remains a major cause of maternal morbidity. This study aimed to describe the clinical, ultrasonographic and histopathological features of EP, including changes following methotrexate (MTX) therapy. **Methods:** A retrospective analysis was conducted on 60 patients diagnosed with EP between 2018 and 2024. Clinical characteristics, serum β-hCG (beta-human chorionic gonadotropin) dynamics, treatment type, site-specific ultrasonographic features, and histopathological aspects were evaluated. **Results:** Extrauterine EPs accounted for 63.3% of cases, predominantly tubal ectopic pregnancy (T-EP), while uterine ectopic pregnancy represented 33.3%, including cesarean scar pregnancy (CSP) in 20%. Heterotopic pregnancy was identified in 3.3%. T-EP most frequently required surgical management, whereas MTX was effective in selected T-EP and CSP cases, as demonstrated by a ≥ 15% decline in serum β-hCG levels at 7 days. Transvaginal ultrasonography (TVUS) enabled accurate site-specific localization of ectopic implantation. Histopathological evaluation confirmed ectopic implantation and MTX-related changes in treated cases. **Conclusions:** Integrating clinical findings, β-hCG dynamics, and targeted TVUS allows accurate diagnosis and individualized management of EP, with histopathology providing definitive confirmation and insight into treatment-related changes.

## 1. Introduction

Ectopic pregnancy (EP) represents a severe obstetric complication, accounting for approximately 2% of all pregnancies and remaining a potentially life-threatening condition, particularly in settings with delayed diagnosis or limited access to medical care [1]. Under physiological conditions, the blastocyst implants within the endometrial cavity; however, when implantation occurs elsewhere, an EP develops. Although the classic triad—abdominal pain, vaginal bleeding, and amenorrhea—may raise clinical suspicion, ultrasonography remains the primary tool for accurate localization of the pregnancy and plays a key role in guiding minimally invasive interventions [2].

Extrauterine ectopic pregnancy (Ex-EP) may occur in several anatomical regions, each with distinct characteristics and frequencies. Tubal ectopic pregnancy (T-EP), accounting for approximately 95% of cases, involves implantation of the gestational sac within the fallopian tube. Interstitial ectopic pregnancy (I-EP), representing 2–4% of cases, results from implantation in the interstitial segment of the fallopian tube, passing through the myometrial wall at the uterine fundus and often presenting later in pregnancy. Cesarean-scar pregnancy (CSP), with an incidence below 1%, develops within the anterior uterine wall at the site of a previous cesarean section (CS) and, despite generally favorable treatment outcomes, carries an elevated risk of complications [3,4]. Heterotopic pregnancy (HP), found in 1–3% of cases, is characterized by the co-existence of intrauterine and Ex-EP and may pose considerable management challenges when preservation of the intrauterine pregnancy is desired [5]. Cervical ectopic pregnancy (C-EP), which is extremely rare (<1%), is defined by implantation within the endocervical canal and is frequently associated with prior dilation and curettage (≈70%) [6,7]. Ovarian ectopic pregnancy (O-EP), occurring in <3% of cases, is often linked to intrauterine device use [8]. Finally, abdominal pregnancy—approximately 1%—involves implantation within the peritoneal cavity; although exceptionally rare, some cases have progressed to term with the delivery of healthy infants [5].

Rupture of an EP is the leading cause of maternal death in the first trimester, accounting for 9–14% of cases and 5–10% of all pregnancy-related deaths [5]. Clinical manifestations may be nonspecific and be misinterpreted as conditions such as appendicitis, nephrolithiasis, early miscarriage, or abdominal trauma [9]. Up to 18% of women presenting with these symptoms in early pregnancy may actually have an EP, contributing to a high risk of misdiagnosis [10].

Recognized risk factors include previous EP, tubal pathology, pelvic surgery, ascending pelvic infections, tubal surgery, infertility, smoking, age >35 years, pelvic inflammatory disease, endometriosis, congenital reproductive tract anomalies, pregnancy occurring with an intrauterine device (IUD), and assisted reproductive technologies (ARTs). Although IUD users generally have a lower overall risk of EP compared with women not using contraception, more than half (≈53%) of pregnancies occurring in the presence of an IUD are ectopic [10]. In addition, progestin-only contraceptive methods have been reported as a potential risk factor for EP [11,12]. Experimental and clinical studies suggest that high levels of synthetic progestins may impair tubal ciliary activity and reduce tubal peristalsis, thereby delaying embryo transport and increasing the likelihood of extrauterine implantation. This mechanism has been described particularly in users of progestin-only pills, injectable contraceptives, and levonorgestrel-releasing systems, although the absolute risk remains low [13,14]. Patients with one prior EP have a recurrence risk of ≈10%, which increases to >25% after two or more episodes [10].

T-EP is the most common form and is associated with high maternal morbidity and mortality when rupture occurs [5]. In Western countries, approximately 15% of EPs present with rupture, and evidence suggests an increase during the COVID-19 pandemic [15]. HP cases are increasingly encountered due to widespread ART use, with reported rates of 1 in 100 pregnancies following in vitro fertilization (IVF) and 1 in 7000 in ART cycles involving ovulation induction [5].

The increasing use of IVF procedures correlates with higher rates of EP, ranging from 2.1% to 8.6% after embryo transfer, compared with ≈2% in natural conceptions [16]. The risk is influenced by the number of embryos transferred, the use of fresh rather than cryopreserved embryos, and cleavage-stage (day 3) transfer compared with blastocyst (day 5) transfer [12]. Conversely, studies have not demonstrated a significant association between EP and oral contraceptive use, prior miscarriage, emergency contraception failure, CS, or spontaneous pregnancy loss [10].

The World Health Organization (WHO) reports a global increase in CS rates, reaching approximately 21% of all births, which may contribute to the rising incidence of CSP [17].

Standard diagnostic evaluation includes transvaginal ultrasound (TVUS) or transabdominal ultrasonography (TAUS) and monitoring of serum β-hCG (beta-human chorionic gonadotropin) levels. Early and accurate diagnosis is crucial for reducing maternal mortality. Current research focuses on identifying biomarkers and endometrial assessment techniques to improve diagnostic precision. Once diagnosed, management may be medical, surgical, or expectant, with emphasis on fertility preservation.

The objectives of this study are to highlight the main ultrasonographic features of EP—in both common and rare locations, to characterize clinical manifestations, and to emphasize the importance of histopathological examination for definitive diagnosis and identification of cellular changes after methotrexate (MTX) therapy. By integrating clinical data, imaging findings, β-hCG dynamics, and histopathological confirmation within the same cohort, this study aims to provide a comprehensive and practice-oriented overview of EP management, particularly in rare implantation sites.

The graphical abstract illustrates the complete diagnostic and therapeutic pathway for EP addressed in this study, integrating transvaginal ultrasonography for localization, medical and surgical management options, and histopathological examination for definitive diagnosis and assessment of trophoblastic tissue.

## 2. Materials and Methods

This study was designed as an exploratory retrospective observational analysis aimed at describing and comparing clinical characteristics, obstetric and surgical history, β-hCG dynamics, imaging findings, and histopathological features across different EP locations and management strategies.

Rather than testing a single predefined causal hypothesis, the study sought to explore potential associations between patient-related factors (e.g., obstetric history, prior uterine surgery) and EP presentation, location, and treatment approach.

### Study Population and Eligibility Criteria

This retrospective observational study included all consecutive patients diagnosed with EP and hospitalized at the Obstetrics and Gynecology Clinic II and the Surgery Clinic III of the Craiova County Emergency Clinical Hospital between January 2018 and December 2024. Inclusion criteria were as follows: (1) a confirmed diagnosis of EP based on clinical, imaging, and/or histopathological findings; (2) availability of complete clinical records, including β-hCG measurements and TVUS data; and (3) medical and/or surgical management performed within the study institution. Exclusion criteria were as follows: (1) a pregnancy of unknown location without confirmed ectopic implantation; (2) incomplete medical documentation; and (3) cases managed or followed exclusively in other medical institutions.

A total of 60 patients met the inclusion criteria and were included in the final analysis. The study cohort represents all eligible cases during the study period; no sampling strategy was applied.

The research methodology included the analysis of relevant clinical and paraclinical variables selected to evaluate potential correlations between risk factors and the type of EP. Variables of interest included patient age and place of residence, as well as obstetric history (deliveries and abortions), previous surgical interventions, and serum β-hCG levels at diagnosis.

The study was designed as a clinical-imaging, statistical, and histopathological analysis applied to a cohort of patients diagnosed with EP, selected according to the location of implantation.

The study cohort included patients diagnosed with EP, hospitalized, and evaluated at the Obstetrics-Gynecology Clinic II and the Surgery Clinic III of the Craiova County Emergency Clinical Hospital between 2018 and 2024. A comprehensive evaluation of each patient’s clinical profile was performed, including demographic data, obstetric history, prior surgical interventions, and β-hCG levels at diagnosis. Patients were classified into subgroups based on the location of the uterine ectopic pregnancy (U-EP): cornual ectopic pregnancy (Co-EP), I-EP, C-EP, CSP, and Ex-EP, including T-EP, O-EP, and HP.

Clinical continuous data were compiled and plotted using Microsoft Excel 2010, as mean values and standard deviations. Differences between paired clinical continuous measurements were assessed using a paired Student’s t-test, after confirming the assumption of normal distribution. Normality of data distribution was assessed prior to parametric testing using graphical inspection and formal normality testing. Homogeneity of variances was evaluated using the Brown–Forsythe and Bartlett tests, as appropriate, before applying one-way ANOVA. Data were analyzed using one-way ANOVA to compare the four time points. When the ANOVA indicated a significant effect, post hoc pairwise comparisons were performed using Tukey’s honestly significant difference (HSD) test. For all ANOVA analyses, post hoc pairwise comparisons were systematically performed when overall significance was detected, even if only selected comparisons reached statistical significance. For all tests, a significance threshold of *p* < 0.05 was considered. Statistical analyses were performed to explore differences and associations between predefined subgroups rather than to test a single confirmatory hypothesis. For subgroups with small sample sizes, analyses were limited to descriptive statistics only. Inferential statistical tests were not applied to these subgroups due to insufficient statistical power. Accordingly, results for rare ectopic pregnancy subtypes are reported descriptively and interpreted with caution. For variables describing patient demographics (age and place of residence), analyses were primarily descriptive, as the small size of certain EP subgroups did not allow for robust inferential statistical testing. Depending on the therapeutic approach, patients were grouped into those treated medically with MTX and those requiring surgical intervention. All participants were informed about the purpose of the study, provided consent for anonymous participation, and agreed to the scientific use of collected tissue samples. The study was approved by the Ethics Committee of the Craiova County Emergency Clinical Hospital (50161/21 October 2025).

In cases where medical treatment was insufficient and surgical excision was required; the collected tissue fragments were sent to the Department of Pathology of the Craiova County Emergency Clinical Hospital. After histopathologic diagnosis, selected tissue blocks were subsequently forwarded to the Center for Microscopic Morphology and Immunology Studies of the University of Medicine and Pharmacy of Craiova for detailed histological evaluation.

Collected tissues were fixed in 10% neutral buffered formalin at room temperature, then routinely processed for paraffin embedding. The resulting blocks were sectioned at 5 µm using an HM350 microtome (ThermoFischer, Waltham, MA, USA) equipped with a Section Transfer System. Sections were mounted on simple glass slides or on poly-L-lysine–coated slides and dried in an incubator at 37 °C for 24 h.

For histological evaluation, classical Hematoxylin–Eosin (HE) staining was performed. Sections were deparaffinized in three xylene baths (10 min each), followed by progressive rehydration through decreasing ethanol concentrations (100%, 96%, 90%, 70%), and rinsed in distilled water. Hematoxylin was used to stain nuclei, and eosin to stain cytoplasmic structures.

The slides were digitized using a Nikon Eclipse 90i motorized microscope (Nikon Instruments, Europe BV, Amsterdam, The Netherlands), equipped with a Prior ES111 OptiScan motorized stage (Prior Scientific, Cambridge, UK) and a cooled 16-megapixel Nikon DS-Ri-2 CMOS camera (Nikon Instruments Inc., Tokyo, Japan). Image acquisition was performed using Nikon NIS-Elements AR software, version 4.20 (Nikon Instruments Inc., Tokyo, Japan).

## 3. Results

### 3.1. Clinical Findings According to the Type of Ectopic Pregnancy

#### 3.1.1. Distribution of Patients According to the Type of EP

A total of 60 patients diagnosed with EP between 2018 and 2024 were included in the analysis. Ex-EP accounted for 38 cases (63.33%), U-EP for 20 cases (33.33%), and HP for 2 cases (3.33%).

**Figure 1:**
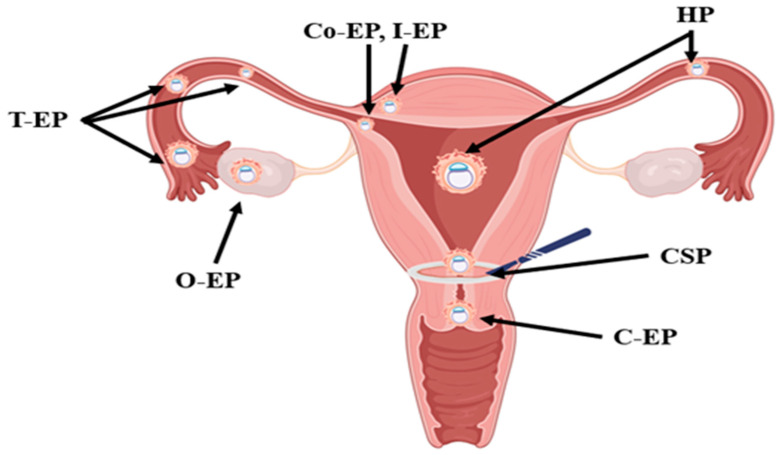
Schematic representation of the types of ectopic pregnancy (EP) included in the study. EP: ectopic pregnancy; T-EP: tubal ectopic pregnancy; O-EP: ovarian ectopic pregnancy; Co-EP: cornual ectopic pregnancy; C-EP: cervical ectopic pregnancy; CSP: cesarean scar pregnancy; I-EP: interstitial ectopic pregnancy; HP: heterotopic pregnancy. Created in Created with BioRender.com (BioRender, Toronto, ON, Canada). Istrate-Ofițeru, A. (2025) BioRender.com/0rmb2o8.

**Figure 2:**
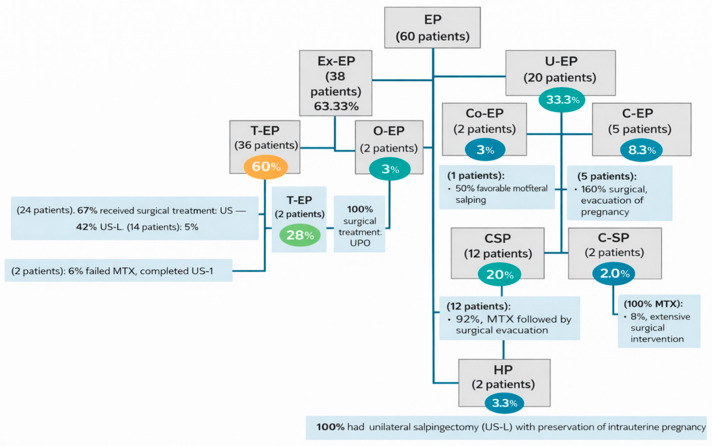
Distribution of ectopic pregnancy (EP) cases according to anatomical location and therapeutic management. The study cohort included 60 patients diagnosed with EP. Extrauterine ectopic pregnancies (Ex-EP) comprised tubal ectopic pregnancy (T-EP) and ovarian ectopic pregnancy (O-EP), while uterine ectopic pregnancies (U-EP) included cornual ectopic pregnancy (Co-EP), cervical ectopic pregnancy (C-EP), cesarean scar pregnancy (CSP), and interstitial ectopic pregnancy (I-EP). Treatment modalities included medical management with methotrexate (MTX) and surgical interventions, selected according to clinical presentation and hemodynamic status. Percentages are reported relative to the total number of ectopic pregnancy cases. EP: ectopic pregnancy; Ex-EP: extrauterine ectopic pregnancy; U-EP: uterine ectopic pregnancy; T-EP: tubal ectopic pregnancy; O-EP: ovarian ectopic pregnancy; Co-EP: cornual ectopic pregnancy; C-EP: cervical ectopic pregnancy; CSP: cesarean scar pregnancy; I-EP: interstitial ectopic pregnancy; HP: heterotopic pregnancy; MTX: methotrexate; US-L: unilateral salpingectomy via laparotomy; US-l: unilateral laparoscopic salpingectomy; UPO: unilateral partial oophorectomy.

Within the Ex-EP group, T-EP was predominant (36 cases, 60%), followed by O-EP (2 cases, 3%). The U-EP group included 12 CSPs (20%), 5 C-EPs (8%), 2 Co-EPs (3%), and 1 I-EP (2%) (Figure 1 and Figure 2).

#### 3.1.2. Classification of Patients Included in the Study According to the Type of Treatment Selected

Among patients with T-EP, 24 (67%) underwent surgical treatment. Emergency US-L was required in 14 cases (58%) due to hemoperitoneum, while 10 patients (42%) underwent US-l. Medical management with MTX was initially selected in 10 patients (28%), of whom 2 (6%) required subsequent surgical intervention due to treatment failure.

All patients diagnosed with C-EP required surgical evacuation. Among the 12 CSP cases, 11 patients (92%) were managed with MTX followed by ultrasound-guided aspirational evacuation, while one patient (8%) required extensive surgical intervention (total hysterectomy) after refusing pregnancy termination. In this case, intraoperative findings revealed deep placental invasion at the level of the cesarean scar, extending through the uterine wall to the serosa and urinary bladder, consistent with placenta accreta spectrum, most likely placenta percreta. The two Co-EP cases evolved differently, one showing favorable progression toward the uterine cavity and one resulting in spontaneous evacuation.

Clinical presentation at admission was variably documented due to the retrospective nature of the study. The presence of the classical EP triad—abdominal pain, vaginal bleeding, and amenorrhea—was not systematically recorded in all medical files. However, review of available clinical notes indicated that abdominal pain and/or vaginal bleeding were the most frequently reported presenting symptoms, whereas a complete classical triad was inconsistently documented. For this reason, symptom distribution is reported descriptively and was not subjected to formal statistical analysis.

#### 3.1.3. Variations in the Age of the Patients Included in the Study

The following results are presented descriptively due to the limited number of cases in several EP subgroups.

Among patients with T-EP, those managed surgically tended to be older than those successfully treated with MTX. Higher ages were generally observed in more complex or rare EP subtypes, whereas lower mean ages were noted in medically managed T-EPs. Detailed age distributions by EP subtype are summarized in Supplementary Table S1.

#### 3.1.4. The Place of Residence of the Patients Included in the Study

The following results are presented descriptively due to the limited number of cases in several ectopic pregnancy subgroups. Residence data (urban vs. rural) are reported descriptively. Overall, a higher proportion of patients originated from urban areas compared with rural areas. This distribution likely reflects differences in access to medical care and diagnostic facilities, including earlier availability of transvaginal ultrasonography and β-hCG testing in urban settings. No inferential statistical analyses were performed due to subgroup size limitations. Detailed distributions of place of residence by ectopic pregnancy subtype are provided in Supplementary Table S1.

#### 3.1.5. Obstetric History of the Patients Included in the Study

The following results are presented descriptively due to the limited number of cases in several EP subgroups. A substantial proportion of patients had a history of prior obstetric events, particularly CS, which was more frequently observed among uterine EP subtypes such as CSP and C-EP. Previous vaginal deliveries and abortions were variably distributed across EP subtypes. Given the heterogeneity of the cohort and the limited size of several subgroups, these findings are reported descriptively without formal statistical testing. Detailed obstetric history data by ectopic pregnancy subtype are summarized in Supplementary Table S1.

#### 3.1.6. Gynecological Surgical History of the Patients Included in the Study

The following results are presented descriptively due to the limited number of cases in several ectopic pregnancy subgroups. Previous gynecological surgical interventions were most frequently observed among patients with T-EP and O-EP, whereas no prior gynecological surgery was reported in patients with cornual, I-EP, or C-EP. Laparoscopic procedures were more common than open surgical approaches. Due to the limited size of several subgroups, these observations are reported descriptively without inferential statistical analysis. A detailed overview of prior gynecological surgical history by EP subtype is provided in Supplementary Table S1.

#### 3.1.7. Variations in β-hCG Levels According to the Type of Ectopic Pregnancy

Among the patients with T-EP, all 10 patients who underwent US-l had minimal hemoperitoneum and opted for this approach. Their initial hCG values ranged from 1100 to 3100 mIU/mL, with a mean value of 2152 mIU/mL (±591.56 mIU/mL). In the 14 patients treated with US-L, massive hemoperitoneum was identified on ultrasound, and hCG values ranged from 1560 to 3400 mIU/mL, with a mean value of 2695 mIU/mL (±630.05 mIU/mL).

In patients for whom MTX therapy was attempted, the initial hCG values ranged from 1580 to 2100 mIU/mL, with a mean value of 1840 mIU/mL. At 48 h post-MTX, hCG levels ranged from 1640 to 2240 mIU/mL (mean 1940 mIU/mL), and at day 4, from 1720 to 2340 mIU/mL (mean 2030 mIU/mL), showing slight increases. By day 7, values ranged from 1910 to 2560 mIU/mL (mean 2235 mIU/mL), without achieving the ≥15% decrease required for treatment success; thus, patients opted for US-l.

**Figure 3:**
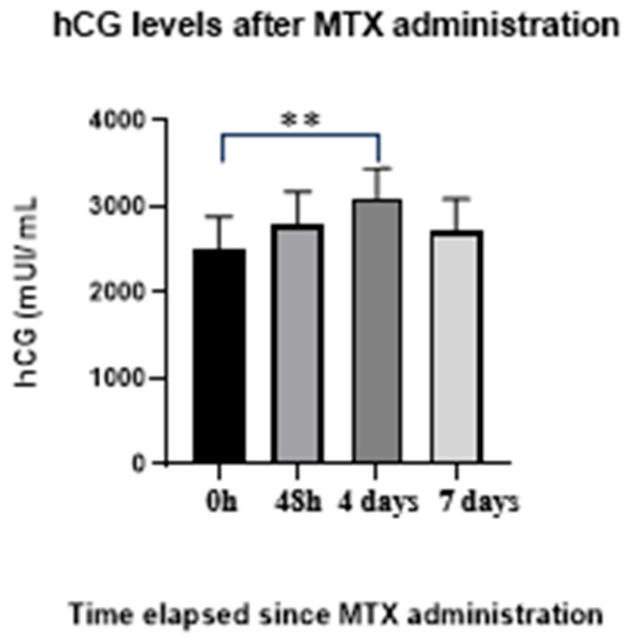
Temporal evolution of serum β-hCG levels at 0 h, 48 h, 4 days, and 7 days after MTX administration. Individual data points and group means are shown for each time point. One-way ANOVA demonstrated significant differences across time (F(3,36) = 4.522, *p* = 0.0086). Tukey’s post hoc test was applied to all pairwise comparisons; a significant difference was identified only between baseline (0 h) and day 4 (adjusted *p* = 0.0046), indicating a peak effect at day 4, followed by a partial decline at day 7. Error bars represent standard deviation. ** indicates *p* < 0.01; MTX: methotrexate.

In another 10 patients treated with MTX, initial hCG values ranged from 2100 to 3100 mIU/mL (mean 2506 mIU/mL ± 383.7 mIU/mL). At 48 h, values ranged from 2300 to 3240 mIU/mL (mean 2801 mIU/mL ± 377.69 mIU/mL). At day 4, values ranged from 2600 to 3450 mIU/mL (mean 3106 mIU/mL ± 345.32 mIU/mL). At day 7, values ranged from 2200 to 3420 mIU/mL (mean 2724 mIU/mL ± 368.63 mIU/mL), showing a >15% decrease compared with the previous measurement. Serum β-hCG levels exhibited significant temporal variation following MTX administration. Mean values increased progressively from baseline (0 h) to 48 h and reached their highest level at day 4, followed by a partial decline by day 7. One-way ANOVA confirmed that these differences across time points were statistically significant (F(3,36) = 4.522, *p* = 0.0086). No significant differences were observed between 0 h and 48 h, 48 h and 4 days, or 4 and 7 days. Post hoc pairwise comparisons were performed for all time points using Tukey’s honestly significant difference (HSD) test. A statistically significant difference was observed only between baseline (0 h) and day 4 (adjusted *p* = 0.0046), indicating a peak effect at day 4 after MTX administration. No statistically significant differences were identified between baseline and 48 h, between 48 h and day 4, or between day 4 and day 7. Overall, these findings demonstrate a delayed peak in β-hCG levels at day 4, followed by a partial decline by day 7 (Figure 3).

Among patients with O-EP, hCG values ranged from 2870 to 2970 mIU/mL, with a mean of 2920 mIU/mL. Due to the presence of moderate hemoperitoneum, unilateral partial oophorectomy (UPO) via laparotomy was performed.

In patients with HP, hCG values were >50,000 mIU/mL, consistent with the normal evolution of an intrauterine pregnancy.

In patients with Co-EP showing favorable normal progression toward the uterine cavity (NP), the initial hCG values ranged from 3210 to 4560 mIU/mL, with a mean value of 3885 mIU/mL. At 48 h, hCG values ranged from 5600 to 9200 mIU/mL (mean 7400 mIU/mL). At 4 days, values ranged from 11,300 to 19,300 mIU/mL (mean 15,300 mIU/mL), and at 7 days, values ranged from 23,000 to 41,000 mIU/mL (mean 32,000 mIU/mL). These findings indicate a gradual, physiologic increase in hCG levels, consistent with a favorable NP.

In the case of I-EP, hCG values increased progressively from 3100 to 3900, then to 4500 and 5000 mIU/mL upon serial assessment, leading to the decision to perform laparoscopic surgery using Argon Plasma Coagulation (APC).

In patients with C-EP, initial hCG values ranged from 2340 to 4120 mIU/mL, with a mean value of 3346 mIU/mL (±740.32 mIU/mL). Values increased significantly over time, reaching 5100–8230 mIU/mL at 48 h, with a mean of 6852 mIU/mL (±148730 mIU/mL), and showed a statistically significant difference, t(8) = –4.584, *p* < 0.001.

**Figure 4:**
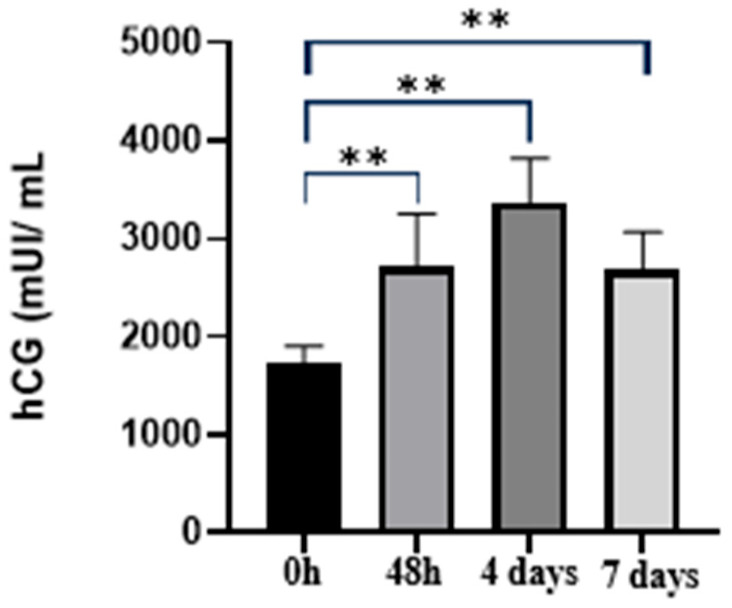
Temporal evolution of serum β-hCG levels at 0 h, 48 h, 4 days, and 7 days after MTX administration in patients with cesarean scar pregnancy. Individual data points and group means are displayed for each time point. One-way ANOVA demonstrated a significant overall effect of time (F(3,40) = 28.98, *p* < 0.0001). Tukey’s post hoc test was applied to all pairwise comparisons. Baseline values were significantly lower than all subsequent measurements (*p* < 0.0001), with a peak observed at day 4. The day 4 values were significantly higher than those at 48 h (*p* = 0.0039) and 7 days (*p* = 0.0019), whereas no significant difference was observed between the 48 h and 7-day measurements (*p* = 0.9948). Error bars represent standard deviation. ** indicates *p* < 0.01; MTX: methotrexate; CSP: cesarean scar pregnancy.

In patients with CSP treated with MTX followed by surgical evacuation, initial hCG values ranged from 1540 to 2100 mIU/mL, with a mean of 1740.9 mIU/mL (±162.81 mIU/mL). At 48 h, values ranged from 2100 to 3900 mIU/mL (mean 2719.09 mIU/mL ± 534.04 mIU/mL). At 4 days, values ranged from 3000 to 4200 mIU/mL (mean 3360 mIU/mL ± 457.97 mIU/mL). At 7 days after MTX administration, hCG values ranged from 2350 to 3400 mIU/mL, with a mean of 2676.36 mIU/mL (±391.72 mIU/mL), showing a >15% decrease compared with the previous measurement. The parameter measured across the four time points after MTX administration showed a robust and statistically significant temporal progression. Values increased sharply from baseline (0 h) to 48 h and reached their highest levels at 4 days, followed by a partial decline by day 7. One-way ANOVA confirmed significant differences among time points (F(3,40) = 28.98, *p* < 0.0001, R^2^ = 0.6849). Tukey’s post hoc analysis revealed that baseline values (0 h) were significantly lower than all subsequent measurements at 48 h, 4 days, and 7 days (all *p* < 0.0001). The peak response at 4 days was significantly higher than both 48 h values (*p* = 0.0039) and 7-day values (*p* = 0.0019). No significant difference was observed between the 48 h and 7-day time points (*p* = 0.9948). Overall, these findings demonstrate a strong rise in serum β-hCG levels from baseline to day 4 post-MTX, followed by a moderate reduction by day 7 (Figure 4).

In the case of the patient with a CSP who declined pregnancy termination and continued the pregnancy until 36 weeks—at which point surgical intervention was required, and total hysterectomy was performed due to complications related to placental accreta—the hCG value at diagnosis was greater than 50,000 mIU/mL.

Taken together, these findings highlight the complementary role of clinical characteristics, β-hCG dynamics, and treatment response in EP. Differences observed across EP locations and management strategies were further correlated with site-specific ultrasonographic patterns and confirmed by intraoperative and histopathological findings, as detailed in the following sections.

### 3.2. Ultrasound Findings According to the Type of Ectopic Pregnancy

#### 3.2.1. Ultrasound Findings Identified in Tubal Ectopic Pregnancy

**Figure 5:**
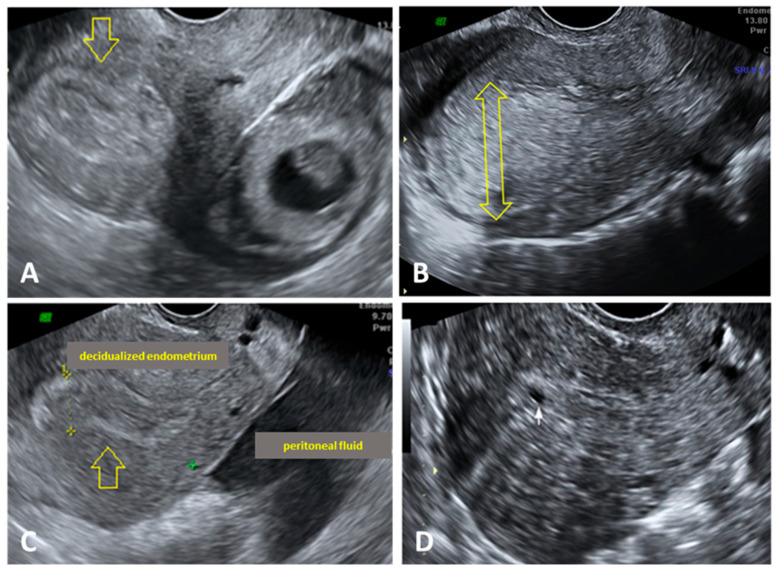
Ultrasound aspects of T-EP. (**A**) A round lateral-uterine mass is visualized, with a gestational sac and embryo confirming T-EP. Endometrial decidual reaction characterized by diffuse endometrial thickening >8 mm with a hyperechoic appearance (yellow arrow); (**B**) Endometrial decidual reaction characterized by diffuse endometrial thickening >8 mm with a hyperechoic appearance (yellow double-headed arrow); (**C**) Presence of retroperitoneal fluid, suggesting blood accumulation in the Douglas pouch, is noted. Endometrial decidual reaction characterized by diffuse endometrial thickening >8 mm with a hy-perechoic appearance (yellow arrow). (**D**) A decidual pseudogestational sac was also detected (white arrow). T-EP: tubal extrauterine pregnancy.

In T-EP, a complex, mobile adnexal mass was observed in the absence of an intrauterine pregnancy, separate from the ovarian structure, with a heterogeneous appearance corresponding to the “blob sign,” and showing peripheral vascularization (Figure 5A). The endometrium may show a decidual reaction with thickening (Figure 5B). In some cases, an empty gestational sac—the “bagel sign”—was identified, while in others, the yolk sac or even an embryo was visualized. Hemoperitoneum was also identified in several cases, potentially due to tubal rupture or blood leakage from the distal tubal end (Figure 5C). In certain cases, a decidual pseudogestational sac was also detected (Figure 5D).

#### 3.2.2. Ultrasound Findings Identified in O-EP

**Figure 6:**
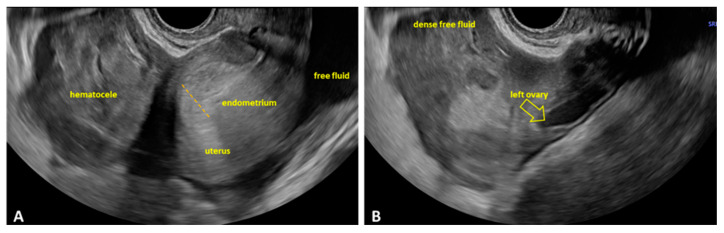
TVUS during emergency department evaluation in a case of O-EP. (**A**) Uterus surrounded by peritoneal fluid, with clear free fluid posteriorly and a complex mass with coarse echoes anterior to the uterus, suggestive of a hematocele; the thickened, decidualized endometrium is visible. (**B**) Clear free fluid and dense free fluid surrounding the uterus, indicating a significant amount of hemoperitoneum. O-EP: ovarian ectopic pregnancy.

In O-EP, TVUS revealed the presence of the pregnancy within the ovarian structure, without the possibility of separating it from the ovary during gentle probe pressure. The trophoblastic tissue generally appeared more hyperechoic than the corpus luteum. On Doppler examination, the corpus luteum is more easily identified, and an additional area of ovarian hypervascularization becomes evident, corresponding to the peritrophoblastic blood flow characteristic of O-EP. Ideally, TVUS demonstrates a gestational sac located within the ovary, while the uterine cavity appears empty. When internal bleeding is present, it may be visualized as a pelvic hematocele or extending into the abdominal cavity. Accurate assessment is crucial to differentiate O-EP from T-EP or from a ruptured ovarian cyst (Figure 6A,B).

#### 3.2.3. Ultrasound Findings Identified in HP

**Figure 7:**
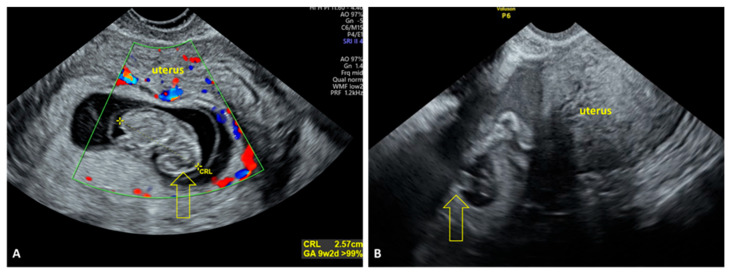
TVUS findings in HP. (**A**) An intrauterine gestational sac with a viable embryo is visualized, with a crown–rump length (CRL) corresponding to 9 weeks and 2 days (yellow arrow). (**B**) A heterogeneous adnexal mass is visualized, containing a gestational sac with a visible yolk sac and free fluid lateral to the ovary (yellow arrow). TVUS: transvaginal ultrasound; HP: heterotopic pregnancy.

In HP, ultrasound findings may vary, but the key indicator is the visualization of two or more gestational sacs—one intrauterine and one or more extrauterine. On TVUS, a normal intrauterine gestational sac with a visible embryo was identified, along with an extrauterine gestational sac located in the tube. Cardiac activity was present in the typically implanted pregnancy. Free peritoneal fluid was also detected, indicating possible internal bleeding due to rupture of the EP (Figure 7A,B).

#### 3.2.4. Ultrasound Findings Identified in I-EP

**Figure 8:**
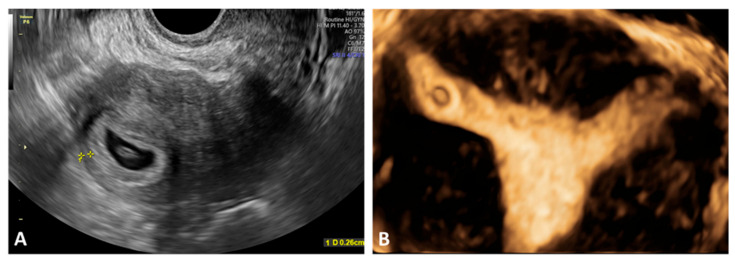
TVUS findings in I-EP. (**A**) Visualization of an I-EP located toward the right uterine border, with preservation of the interstitial line and a myometrial thickness of less than 5 mm (measured using yellow calipers), as well as absence of a gestational sac within the uterine cavity. (**B**) Three-dimensional ultrasound reconstruction highlighting the lateral implantation site at the uterine cornu/interstitial portion, confirming the diagnosis of I-EP. I-EP: interstitial ectopic pregnancy.

Typical TVUS findings in I-EP included the following: an eccentrically located gestational sac positioned laterally in the uterus, at the level of the uterine cornu/interstitial portion of the tube, appearing outside the central endometrial cavity; the “interstitial line sign,” extending from the central endometrial cavity to the gestational sac, a characteristic sonographic marker of I-EP; a thin myometrial mantle surrounding the sac, less than 5 mm in thickness, indicating implantation at the uterine margin rather than within the cavity; absence of a normal intrauterine sac, with an empty central endometrial cavity—critical for differentiating I-EP from an intrauterine pregnancy; a complex mass or heterogeneous echoes in the uterine cornu, which may appear as an irregular, heterogeneous lesion with hypo- or hyperechoic content, sometimes containing a visible sac, other times appearing only as a suspicious mass; and free pelvic fluid suggesting hemoperitoneum, particularly relevant when rupture is suspected (Figure 8A,B).

#### 3.2.5. Ultrasound Findings Identified in C-EP

**Figure 9:**
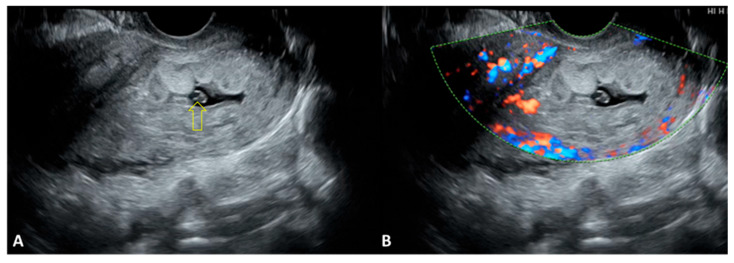
Visualization of a C-EP. (**A**) The gestational sac (yellow arrow) is identified within the cervical canal, producing the characteristic “hourglass” or “barrel-shaped cervix” appearance. (**B**) Color Doppler shows an intense vascular ring surrounding the sac and trophoblast (peritrophoblastic flow), confirming trophoblastic viability and cervical implantation (“ring of fire”). C-EP: cervical ectopic pregnancy.

Characteristic TVUS findings in C-EP showed the presence of a gestational sac within the cervical canal, below the level of the internal os, with an empty uterine cavity and an enlarged cervix (“barrel-shaped cervix”), producing the classic “hourglass” or “barrel” appearance. The sac was generally well defined, oval or round, and entirely located within the cervical canal. With gentle pressure from the transvaginal probe, the sac did not move (“absent sliding sign”), in contrast to the sac observed in spontaneous pregnancy loss (Figure 9A). Color Doppler frequently demonstrated an intense vascular ring surrounding the sac (peritrophoblastic flow), confirming trophoblastic viability and cervical implantation (“ring of fire”) (Figure 9B).

The uterine cavity was empty, with no identifiable sac or embryo, and the endometrium appeared thin and inadequate to support an intrauterine pregnancy.

#### 3.2.6. Ultrasound Findings Identified in CSP

**Figure 10:**
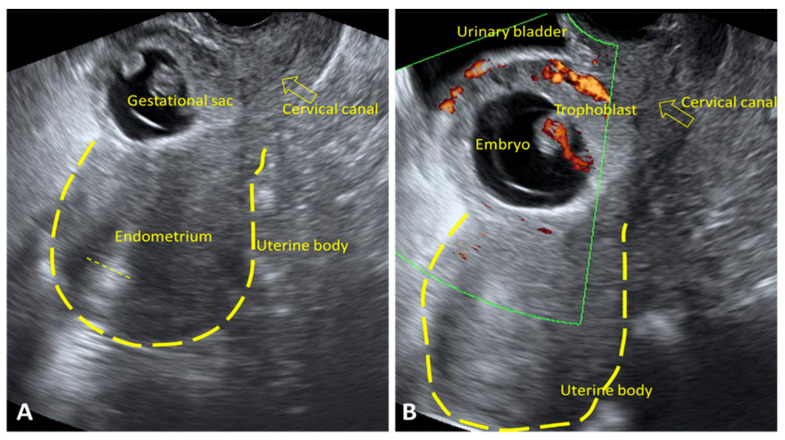
TVUS findings in CSP. (**A**,**B**) The gestational sac is visualized within the post-cesarean section scar niche. The myometrial thickness between the gestational sac and the bladder is minimal, and vascularization is markedly increased. TVUS: transvaginal ultrasound; CSP: cesarean scar pregnancy.

In CSP, the following TVUS features were identified: the uterine body was empty, while the gestational sac was visualized in the lower anterior uterine segment at the level of the cesarean scar. The sac was implanted within the scar niche, immediately anterior to the uterus, distinguishing it from a normal intrauterine pregnancy. The myometrium at this level was very thin or absent between the sac and the bladder (<5 mm), or even completely lacking, highlighting scar implantation. Intense periplacental Doppler flow was also observed, demonstrating marked vascularization around the sac (“ring of fire”) and indicating active trophoblastic proliferation (Figure 10A,B). The shape of the sac (triangular or oval) reflected its accommodation within the scar defect; it appeared “pressed” into the niche, and depending on the position of the sac’s center relative to the endometrial line (“Cross-over sign”), placement below the line indicated true scar implantation.

When the myometrium is extremely thin (<2 mm), there is a high risk of early rupture and massive hemorrhage. Free pelvic fluid suggests hemoperitoneum in complicated cases. Importantly, after 7 weeks of gestation, the sac may appear to “migrate” toward the uterine cavity, but its vascular attachment to the scar persists.

**Figure 11:**
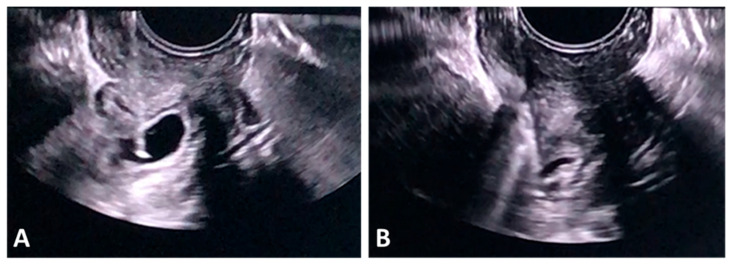
Transvaginal ultrasound (TVUS) findings in cesarean scar pregnancy (CSP) during methotrexate (MTX) treatment. (**A**) TVUS image showing local injection of methotrexate into the gestational sac implanted at the cesarean scar level, with focal expansion of the sac. (**B**) TVUS image obtained after MTX injection, demonstrating transient enlargement of the gestational sac and changes in local vascularization during follow-up. TVUS: transvaginal ultrasound; CSP: cesarean scar pregnancy; MTX: methotrexate.

In the CSP cases included in this study, treatment consisted of MTX administration followed by vacuum aspiration of the gestational tissue. Under ultrasound guidance, 50 mg/m^2^ MTX (diluted in ~5 mL saline) was injected directly into the sac. A localized expansion of the sac was noted, with small bubbles or localized fluid accumulation. Following injection, the sac showed transient enlargement and changes in local vascularization. Continuous monitoring was performed for any signs of bleeding or sac disruption (Figure 11A,B). Subsequently, β-hCG levels were monitored. Once β-hCG decreased to ~10,000 mIU/mL or less, ultrasound-guided aspirational curettage was performed, sometimes with a Foley balloon tamponade for hemostasis. Clinical examination alone does not provide sufficient anatomical detail and relies entirely on ultrasound findings.

In the patient who desired pregnancy preservation and accepted all associated risks, the placenta invaded the uterine wall at the level of the scar, extending to the urinary bladder.

### 3.3. Intraoperative Macroscopic Findings According to the Type of EP

#### 3.3.1. Intraoperative Macroscopic Findings Identified in T-EP

**Figure 12:**
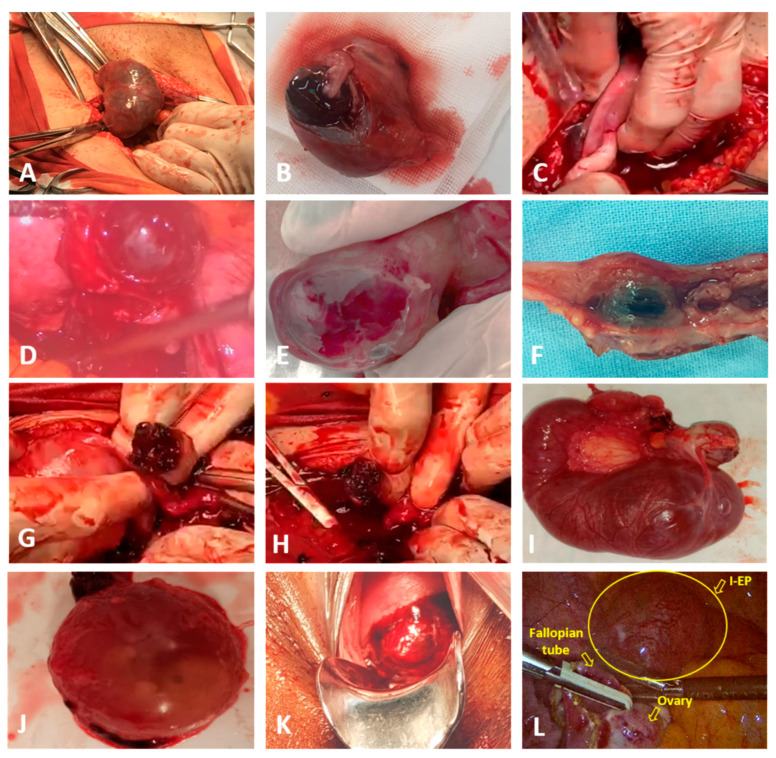
Intraoperative and Postoperative Macroscopic Aspects of EP According to Localization. (**A**) T-EP—The affected tube appears enlarged, measuring approximately 30–40 mm, dilated, congested, with an edematous or friable appearance. (**B**) Minimal dissection reveals a tubal hematoma (hematosalpinx) with visible trophoblastic tissue inside. The external tubal surface shows subserosal hemorrhage and dilated veins. (**C**) The right tube is markedly enlarged, dilated, congested, friable, and actively bleeding, with a large amount of free hematic fluid in the pelvis. (**D**) Visible trophoblastic tissue can be observed at the site of tubal rupture, with hemoperitoneum identified during laparoscopy. (**E**,**F**) Intact gestational sac identified within the tube, with a visible embryo. (**G**) O-EP—Exteriorization of the left adnexa shows a normal left fallopian tube and a left ovary with a violaceous, excentric mass covered by adherent clots and a large crater-shaped defect. (**H**) Massive hemoperitoneum is present, with a breach in the left ovary and adherent clots extending into the pelvic hematoma. (**I**) HP—Macroscopic appearance of T-EP after salpingectomy, showing a markedly dilated tube with a hemorrhagic mass and a gestational sac visible by transparency. (**J**) Gestational sac after tubal dissection, containing an embryo with CRL corresponding to 8 weeks of gestation. (**K**) C-EP—Gestational sac implanted in the cervix, presenting as a vascularized reddish-violaceous mass within the cervical canal, suggesting deep implantation. Active bleeding is visible from the friable cervical surface, with adherent clots. (**L**) I-EP—Intraoperative appearance shows an eccentric, bulging area toward the uterine horn containing chorionic villi and clots, with marked surrounding congestion, focal myometrial rupture, and blood extravasation into the peritoneal cavity. EP: ectopic pregnancy; T-EP: tubal ectopic pregnancy; O-EP: ovarian ectopic pregnancy; HP: heterotopic pregnancy; CRL: crown–rump length; C-EP: cervical ectopic pregnancy; I-EP: interstitial ectopic pregnancy.

During US-l or US-L, T-EP showed characteristic tubal alterations, including enlargement, congestion, and an edematous or friable appearance. The affected segment (usually the ampullary portion) was dilated and contained a tubal hematoma (hematosalpinx), with visible trophoblastic tissue or a gestational sac. The external surface of the tube displayed areas of subserosal hemorrhage and dilated veins, and in some cases, the gestational sac protruded through the tubal wall (Figure 12A,B).

In advanced cases, tubal rupture was present, with active bleeding into the peritoneal cavity (hemoperitoneum), sometimes in large quantities (up to liters in severe cases), as well as clotted blood in the peritoneal spaces (Douglas pouch, between intestinal loops). Gestational tissues (chorionic villi, clotted blood) were often visible at the rupture site or within the pelvic cavity (Figure 12C,D). The peritoneum appeared irritated and sometimes fibrinous; the ovaries were generally normal but covered with blood; the contralateral tube, when present, appeared normal in all cases included in the study.

In rare cases, the gestational sac remained intact within the tube, typically in early, uncomplicated forms (Figure 12E,F).

#### 3.3.2. Intraoperative Macroscopic Aspects Identified in O-EP

In O-EP, intraoperative evaluation revealed the ovary containing a well-defined hemorrhagic mass measuring 2–5 cm, with the appearance of a cystic formation filled with blood. The ovarian surface was disrupted, with active bleeding and adherent clotted blood surrounding the lesion. Trophoblastic tissue was observed within the ovarian cortex (Figure 12G).

These cases were frequently associated with significant hemoperitoneum, reaching up to nearly 1000 mL of blood due to rupture of the ovarian lesion, with free blood and clots in the Douglas pouch and between intestinal loops (Figure 12H).

A key diagnostic element was the presence of an intact ipsilateral fallopian tube, with a normal appearance, as well as normal contralateral adnexa—features that help differentiate O-EP from T-EP. Nevertheless, O-EP may be misinterpreted intraoperatively as a ruptured hemorrhagic cyst, ruptured corpus luteum, hemorrhagic luteinic cyst, or hemorrhagic ovarian tumor; therefore, histopathological confirmation is essential for definitive diagnosis.

#### 3.3.3. Intraoperative Macroscopic Findings Identified in HP

In HP, intraoperative findings demonstrated the presence of a viable intrauterine pregnancy, previously confirmed preoperatively by ultrasound. During surgery, the uterus appeared enlarged for the reported gestational age and markedly congested.

The extrauterine lesions suggestive of HP identified in the cases included in this study were located at the level of the fallopian tube, which appeared dilated and contained a hemorrhagic mass or a visible gestational sac (Figure 12I,J). Active bleeding and peritubal clots were present, consistent with hemoperitoneum. The ovaries and the contralateral tube displayed a normal appearance, while the peritoneum was irritated secondary to intraperitoneal bleeding.

#### 3.3.4. Intraoperative Macroscopic Findings Identified in C-EP

In C-EP, macroscopic findings during surgical evacuation revealed a markedly enlarged, globular, and soft cervix, displaying a violaceous or cyanotic coloration consistent with severe congestion. An abnormal widening of the cervical canal was also observed. In several cases, gestational tissue was visible protruding into the cervical canal or even exteriorized through the vagina (Figure 12K).

During curettage, significant bleeding frequently occurred, disproportionate to the apparent amount of evacuated tissue. The implantation site appeared friable, with abundant bleeding and poor contractility—unlike the uterine cavity—indicating the absence of normal myometrial hemostatic response. Unlike an intrauterine pregnancy, no effective contraction of the cervical wall occurred, which increases the risk of catastrophic hemorrhage.

In the cases included in this study, no additional hemostatic interventions were required. The trophoblastic tissue did not deeply invade or accrete into the cervical wall; no hemoperitoneum developed, and the trophoblast did not extend toward the lower uterine segment or endometrium.

#### 3.3.5. Intraoperative Macroscopic Findings Identified in I-EP

In I-EP developing within the intramural segment of the fallopian tube, the uterus appeared mildly enlarged, with an eccentric, bulging area located near the uterine cornu. On sectioning, a well-defined cavitary focus was identified within the thick myometrium, containing chorionic villi and blood clots. The surrounding tissue was markedly congested, with focal myometrial ruptures and blood extravasation into the peritoneal cavity (Figure 12L).

#### 3.3.6. Intraoperative Macroscopic Findings Identified in CSP

In CSP, inspection of the uterus revealed an abnormal deformation or anterior protrusion at the level of the lower uterine segment. This area appeared thinned and friable, with partial or complete absence of the myometrium between the amniotic sac and the urinary bladder. The uterine wall at the implantation site was extremely thin or translucent (≤2 mm), explaining the high risk of early uterine rupture.

Prominent vascular anastomoses between the uterus and the bladder, as well as large exposed vessels on the uterine surface, were noted in the case in which the patient declined medical treatment and continued the pregnancy until 36 weeks. In this situation, the placenta invaded deeply into the myometrium, resulting in placental accreta and necessitating hysterectomy with bladder repair.

### 3.4. Microscopic Findings Identified According to the Type of EP

#### 3.4.1. Microscopic Findings Identified in T-EP

In T-EP, microscopic examination of the fallopian tube revealed chorionic villi (trophoblastic structures) that were both viable—with well-defined trophoblast and nucleated cells in the villous mesoderm—and degenerated, showing fibrosis, calcifications, and hemorrhage. Trophoblastic invasion of the tubal wall was evident, with cytotrophoblast and syncytiotrophoblast cells infiltrating the muscular and serosal layers. In cases complicated by hemoperitoneum, the trophoblast extended through the full thickness of the tubal wall, reaching the peritoneal surface.

**Figure 13:**
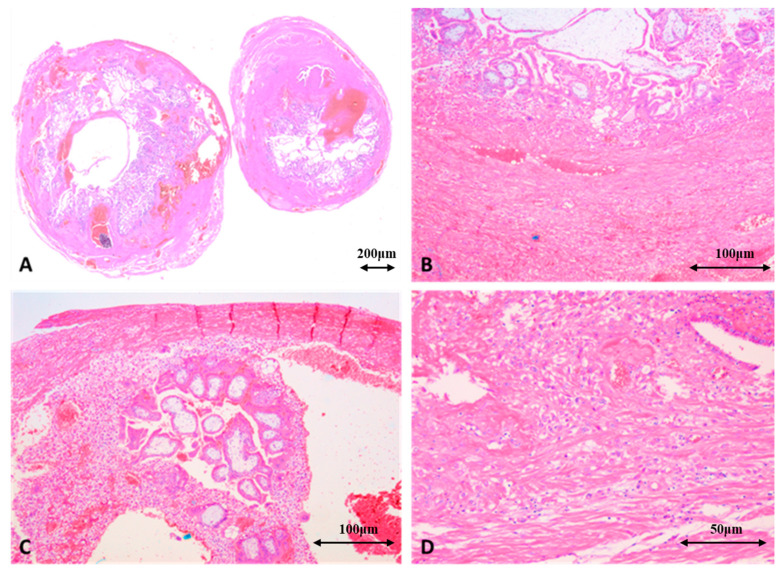
Transverse section of the fallopian tube diagnostic for T-EP. (**A**) Two complete cross-sections of the tube showing trophoblastic invasion of the tubal wall adjacent to the tubal epithelium, with extensive luminal hemorrhage. HE stain, ×40. (**B**,**C**) Transverse sections of the tubal wall infiltrated by chorionic villi, with adjacent edema, focal hemorrhage, and disruption of the local histoarchitecture. HE stain, ×100. (**D**) Trophoblastic cells identified between smooth muscle fibers. HE stain, ×100. HE: Hematoxylin–Eosin; T-EP: tubal ectopic pregnancy.

Additional microscopic findings included intraluminal and extraluminal hemorrhage, acute or chronic inflammatory infiltrates—particularly in cases undergoing tubal abortion—along with degenerative changes in the tube such as edema, focal necrosis, epithelial destruction, and thrombi within tubal vessels (Figure 13A,B). Local inflammation may contribute to long-term sequelae (obstruction, adhesions) in patients who do not undergo salpingectomy.

Histopathological examination is essential for differentiating T-EP from intrauterine trophoblastic tissue (seen in normal pregnancy or miscarriage), from trophoblastic tumors (e.g., hydatidiform mole, characterized by edematous villi and abnormal trophoblastic proliferation), and from chronic inflammatory conditions of the tube (salpingitis) that lack trophoblastic elements.

#### 3.4.2. Microscopic Findings Identified in O-EP

In O-EP, microscopic examination revealed well-defined chorionic villi—composed of mesoderm and trophoblastic cells—embedded directly within the ovarian parenchyma rather than in the tubal lumen. These villi displayed both viable forms, with intact trophoblast, and degenerative forms characterized by fibrosis, edema, or microcalcifications. The adjacent ovarian tissue was infiltrated by trophoblastic cells, with cytotrophoblast and syncytiotrophoblast eroding the normal follicular architecture and accompanied by peritrophoblastic hemorrhage.

**Figure 14:**
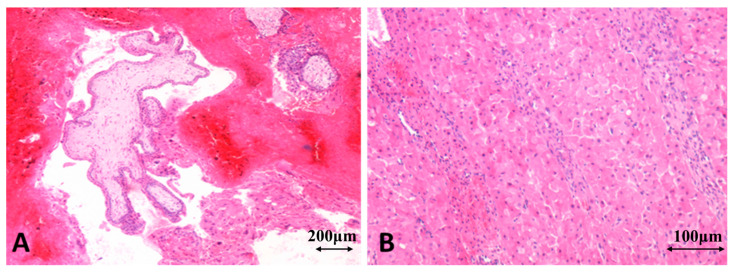
Microscopic features of O-EP. (**A**) Hemorrhagic necrosis with diffuse extravillous trophoblast infiltrating the interstitial spaces, including multinucleated giant trophoblastic cells. Mesenchymal placental villi lined by a biphasic trophoblastic layer are present, composed of an inner layer of cytotrophoblast and an outer layer of syncytiotrophoblast, showing a hypercellular and reduced vascularity. HE stain, ×40. (**B**) Luteinized cells adjacent to chorionic villi, along with peri-luteal and intraluteal lymphoplasmacytic inflammatory infiltrate. HE stain, ×40, ×100.

A constant microscopic feature was the presence of massive intraparenchymal hemorrhage, often simulating a ruptured hemorrhagic cyst or hemorrhagic corpus luteum. Near the implantation site, a corpus luteum was frequently identified, showing large luteinized cells with eosinophilic cytoplasm (Figure 14A,B).

Histopathological diagnosis relied on the modified Spiegelberg criteria, which include intraoperative demonstration of an intact fallopian tube separate from the ovary, localization of the gestational tissue within the ovarian parenchyma, preservation of ovarian–uterine continuity via the utero-ovarian ligament, and the presence of ovarian tissue within the wall of the gestational sac. Differential diagnosis included a hemorrhagic cyst, a ruptured corpus luteum, or a ruptured T-EP with secondary bleeding into the ovary, none of which contain implanted chorionic villi.

#### 3.4.3. Microscopic Findings Identified in HP

In cases where the tubal EP is associated with HP, the microscopic appearance essentially mirrors that of a typical T-EP. However, the diagnosis may become more challenging when the clinical context strongly suggests an isolated intrauterine pregnancy. Within the lumen of the fallopian tube, chorionic villi were identified, occasionally showing early infiltration of the tubal wall. These villi exhibited an immature appearance, with a poorly vascularized mesenchymal core, yet also demonstrated degenerative changes such as edema, fibrosis, or calcifications.

Extravillous trophoblastic invasion was prominent, with cytotrophoblast and syncytiotrophoblast progressively infiltrating the mucosal and muscular layers of the tube. This process resulted in deep tissue destruction and ultimately tubal rupture. Marked hemorrhage—both intratubal and peritubal—was observed, frequently accompanied by areas of hemorrhagic necrosis and capable of producing hemoperitoneum.

The surrounding tissue showed an evident inflammatory response, with lymphoplasmacytic or granulocytic infiltrates, reflecting the local immune reaction to trophoblastic invasion. Histologic examination of the tube did not reveal uterine-type tissue, confirming a tubal implantation site.

Microscopic evaluation is essential for differentiating HP from a solitary T-EP without an associated intrauterine pregnancy, from decidual remnants lacking villi or trophoblast, and from a hemorrhagic tubal cyst, which shows no trophoblastic cells or chorionic villi.

#### 3.4.4. Microscopic Findings Identified in I-EP

In I-EP, microscopic examination demonstrated chorionic villi implanted within the intramural portion of the fallopian tube, located deep in the thickness of the myometrium. The villi were generally well preserved but also showed degenerative changes, including edema, fibrosis, and microcalcifications. Extravillous trophoblastic tissue exhibited an infiltrative pattern, extending between smooth muscle fibers and causing local tissue destruction—a process that explains the high risk of delayed yet severe rupture.

Marked hemorrhage was observed surrounding the implantation site, both within the myometrium and within adjacent tissues, frequently accompanied by areas of hemorrhagic necrosis. The inflammatory response consisted of a lymphocytic or mixed inflammatory infiltrate, indicating the local reaction to trophoblastic invasion.

An examination of the interstitial region confirmed its continuity with the myometrium while showing clear separation from the true tubal lumen—an essential feature for distinguishing an I-EP from an isthmic tubal pregnancy.

#### 3.4.5. Microscopic Findings Identified in C-EP

In C-EP, histopathological examination of the curettage specimen shows distinctive features essential for the differential diagnosis from an intrauterine pregnancy (normal or abortive), mainly because ultrasound may occasionally be inconclusive. Microscopic evaluation of C-EP specimens revealed absent or only scant, superficial, and immature chorionic villi, which were poorly vascularized and often displayed degenerative changes such as edema or fibrosis, accompanied by degenerating trophoblast.

Decidual tissue was atypical or absent: the uterine decidua was poorly developed or completely missing, in contrast to a normal intrauterine pregnancy. This represents an important indirect indicator of C-EP. Reactive alterations and moderate to severe chronic inflammation were also present. Within the endometrium, a pseudodecidual change was identified, but without implantation of villi into the mucosal thickness. The evacuated tissue often showed extensive hemorrhagic areas, consistent with the active bleeding typically associated with C-EP.

Although curettage material in C-EP is not definitive for a positive diagnosis, the absence of well-developed chorionic villi and decidua represents a strong indirect clue that implantation is not intrauterine. The diagnosis requires a combined clinical, imaging, and histopathological assessment, but microscopic analysis can strongly suggest C-EP.

#### 3.4.6. Microscopic Features Identified in CSP

Microscopic evaluation of tissue obtained by aspiration curettage after MTX injection in CSP demonstrated degenerative chorionic villi (a definitive indicator of pregnancy), which were immature, with reduced blood flow, and displayed marked edematous degeneration and fibrinoid necrosis. The villous structures were frequently fragmented or partially destroyed due to MTX’s cytotoxic effect. Both cytotrophoblast and syncytiotrophoblast exhibited clear apoptotic changes, including nuclear condensation and fragmentation, along with atrophy or partial denudation from the villous surface.

Because the implantation occurred outside the endometrium—within the myometrial tissue of the cesarean scar—the aspirated material lacked typical uterine decidua.

**Figure 15:**
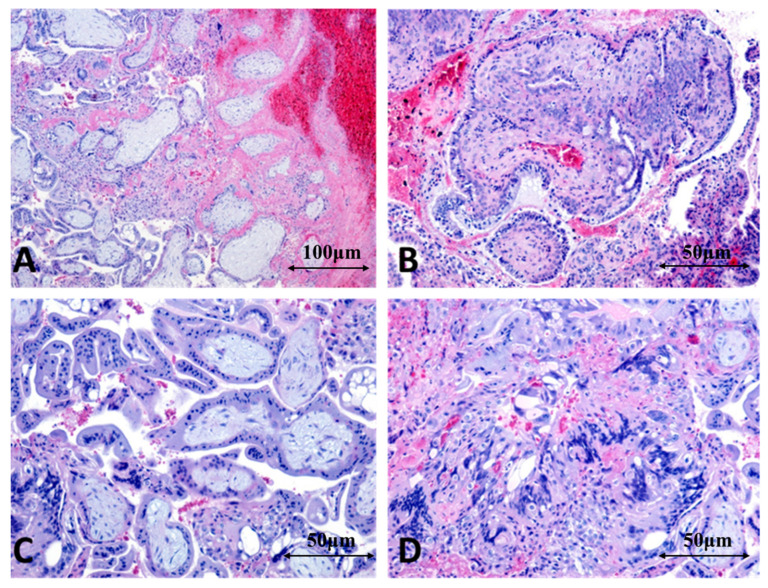
Microscopic features of tissue obtained by aspiration curettage after MTX injection in CSP. (**A**–**C**) Chorionic villi (definitive evidence of pregnancy) are present but appear immature, with reduced blood flow, and show edematous degeneration, fibrinoid necrosis, and frequent fragmentation, with villous structures partially destroyed by the cytotoxic effect of MTX and exhibiting cellular apoptosis. (**C**,**D**) Multinucleated giant cells are also visible, arising secondarily from tissue degradation. Classical HE staining, ×100, ×200. MTX: methotrexate; CSP: cesarean scar pregnancy; HE: Hematoxylin–Eosin.

Trophoblastic remnants were frequently accompanied by recent or old hemorrhage (hematin, hemosiderin deposits), occasional organized thrombi, and fragments of liquefactive necrosis. In some cases, a reactive lymphoplasmacytic inflammatory infiltrate was present, along with multinucleated giant cells formed secondarily to tissue breakdown (Figure 15A–D).

Overall, the aspirated tissue following local MTX administration in CSP reflected complete or partial degeneration of the conceptus, characterized by necrotic chorionic villi and trophoblast undergoing MTX-induced apoptosis. Histopathological examination was essential for confirming the diagnosis and excluding a residual viable intrauterine pregnancy.

## 4. Discussion

### 4.1. Discussion on the Patients’ History and Their Clinical Characteristics in EP

Although the classical triad of EP—abdominal pain, vaginal bleeding, and amenorrhea—is traditionally described in textbooks, multiple studies have shown that only a minority of patients present with all three symptoms simultaneously and reliance on this triad alone may contribute to delayed or missed diagnosis. In our cohort, the complete triad was not consistently documented, reflecting real-world emergency presentations and the retrospective nature of data collection. These findings are consistent with existing literature and underscore the importance of integrating clinical assessment with biochemical markers and imaging findings for timely and accurate diagnosis [9,10,18].

#### 4.1.1. Factors Involved in the Development of Ectopic Pregnancies: General Considerations

EP represents a major reproductive health concern, defined by implantation of the embryo outside the uterine cavity. Although T-EP is the most common form, rarer entities such as O-EP, I-EP, C-EP, HP, and CSP are reported with increasing frequency. This trend has been linked to the rising number of uterine surgical procedures and the widespread use of ART.

T-EP accounts for approximately 95% of cases and is most often related to tubal damage caused by genital infections, particularly Chlamydia trachomatis, chronic salpingitis, or prior tubal surgery. These factors impair ciliary function and tubal peristalsis, delaying embryo transport and facilitating ectopic implantation. Histologically, trophoblastic invasion of the tubal wall leads to tissue destruction and intraperitoneal hemorrhage [14,19].

O-EP is a rare forms of EP, defined by implantation of the conceptus within the ovarian cortex, and its diagnosis relies on Spiegelberg’s criteria [20]. Although its pathogenesis remains incompletely understood, implantation may occur before follicular rupture, particularly in altered ovulatory cycles or IVF [21].

In our cohort, I-EP was associated with prior uterine surgery, consistent with previous reports [22]. Diagnosis is challenging and often delayed, requiring high-resolution ultrasonography and, in some cases, laparoscopy [23].

C-EP, defined by implantation of the blastocyst within the endocervical canal below the internal os, is rare but associated with a high risk of severe hemorrhage. Reported risk factors include repeated curettage, cervical surgical procedures, and ART [24]. Histologically, chorionic villi are identified within cervical tissue, often surrounded by hemorrhage and necrosis, lacking a normal endometrial decidua, underscoring the need for early diagnosis and cautious management [7,24,25].

CSP, is an increasingly recognized form of EP. Implantation occurs within the myometrial defect at the site of a previous cesarean section. Consistent with prior studies, we observed that predisposing factors include multiple CS, short intervals between surgery and conception, and inadequate healing of the uterine incision [26]. Histologically, we identified villous implantation deep within the lower uterine myometrium, without a normal adjacent uterine cavity.

The relatively high proportion of HP, C-EP and CSP observed in our cohort likely reflects referral bias related to the tertiary-care profile of our institution, which serves as a regional referral center for complex and atypical cases. Similar patterns have been reported in other specialized centers [19,27].

In particular, the increased number of CSP is consistent with the rising CS rate and the systematic referral of suspected CSP cases to specialized centers for diagnostic confirmation and management. Similar referral patterns have been described for C-EP and HP, which are associated with significant diagnostic challenges and a high risk of hemorrhagic complications [24].

Importantly, CSP should be regarded as part of a pathological continuum of abnormal placentation rather than as distinct entity. Early implantation within a cesarean scar niche is increasingly recognized as an initial stage of abnormal placentation. If the pregnancy progresses, this abnormal implantation may evolve into placenta previa and subsequently into placenta accreta spectrum, due to deep and progressive trophoblastic invasion of the myometrium at the scar site [28,29].

Several authors have proposed that CSP represents the earliest identifiable form of PAS, particularly in cases where the pregnancy is allowed to continue beyond the first trimester. This concept explains the coexistence or progression from CSP to placenta previa or placenta accreta observed in selected cases, including those reported in our cohort [28,29].

#### 4.1.2. Discussion Regarding Patients’ Age in EP

Maternal age represents a relevant demographic factor in evaluating the risk of EP. Most studies report a higher incidence in women aged 25–34 years, with a further increase after 35 years, likely reflecting cumulative exposure to pelvic pathology and prior uterine or tubal interventions [19]. Large population-based study by Bouyer et al. identified a peak incidence between 30 and 34 years, with a significant age-related risk increase thereafter [19]. In the context of ART, a higher frequency of EP has been observed in patients over 35 years of age, who more commonly undergo IVF [30,31]. Although EP may also occur in adolescents, its incidence is lower and is primarily associated with untreated sexually transmitted infections or limited access to modern contraceptive methods [13]. In contrast, rare forms of EP (O-EP, C-EP, CSP) tend to occur more frequently in women of active reproductive age with complex obstetric histories, particularly following uterine interventions or CS [7,19,24].

Overall, the age distribution observed in our cohort is consisted with these findings and supports the need for increased clinical vigilance in women over 30 years old.

#### 4.1.3. Discussion Regarding the Patients’ Background Environment in EP

The patients’ living environment, whether urban (U) or rural (R), is an important epidemiological factor in EP, reflecting differences in access to medical care, health education, contraceptive methods, and treatment for gynecological infections. Rural populations are more frequently diagnosed at advanced stages, with higher rates of complications such as tubal rupture or hemoperitoneum, largely due to limited access to transvaginal ultrasonography and delayed presentation [19].

In contrast, U settings allow earlier diagnosis through improved access to β-hCG testing and routine TVUS, although a higher proportion of EP related to ART is also reported [31].

While biological factors remain central to EP development, sociodemographic factors influence diagnostic timing and management. In line with this, our cohort showed a higher proportion of cases among patients from urban areas.

#### 4.1.4. Discussion Regarding the Obstetric History of Patients with EP

Obstetric history is a key determinant in EP risk assessment, as prior obstetric events may alter uterine and tubal anatomy and function. CS represents a major risk factor, particularly for CSP, where implantation within a structurally weakened scar may result in severe hemorrhage or early uterine rupture [26].

A history of spontaneous or induced abortion especially when associated with repeated uterine curettage, increases the risk of ectopic implantation, including cervical and cesarean scar locations [24,32]. In addition, post-abortive infections may lead to pelvic inflammatory disease, impair tubal patency, and increase the likelihood of T-EP [19].

Vaginal deliveries (VD) are not directly associated with EP; however, multiparity may serve as an indirect risk factor reflecting cumulative exposure to uterine interventions or gynecological infections [13].

In line with these findings, our study identified a strong association between complex obstetric history—particularly CS and A—and EP, underscoring the importance of detailed obstetric assessment in patients with suspected EP.

#### 4.1.5. Discussion Regarding Past Medical History: Gynecological Surgical Interventions

Previous gynecological surgery represents a major risk factor for EP as surgical interventions may disrupt tubal, endometrial, or myometrial anatomy and function. Procedures such as partial salpingectomy, tubal ligation or recanalization, and surgery for prior EP can compromise tubal epithelial integrity and peristalsis, impairing embryo transport [19,30].

Uterine surgeries, including myomectomy, may lead to scar formation that predisposes to abnormal implantation, including I-EP or CSP. Intrauterine procedures such as repeated curettage or operative hysteroscopy may result in adhesions or inadequate decidualization, favoring implantation at non-physiological sites, including the cervix or fallopian tubes [13].

Previous CS is strongly associated with CSP, with risk increasing with the number of procedures and influenced by surgical technique and the interval to subsequent conception [26].

Overall, a detailed assessment of prior gynecological and obstetric surgeries is essential in patients with suspected EP.

#### 4.1.6. Variations in β-hCG Levels in Patients with EP

Serum β-hCG levels are an essential tool for diagnosing and monitoring EP. In EP, the serum β-hCG levels are rising more slowly, plateauing, or even decreasing [18]. Absolute β-hCG values alone do not reliably indicate pregnancy location, and a single measurement cannot distinguish EP from early intrauterine pregnancy or miscarriage [24,32]. However, an increase of less than 66% over 48 h is strongly suggestive of abnormal gestational progression [33].

Some EP may initially present with near-normal β-hCG levels, necessitating close correlation with ultrasonographic findings and serial measurements. When β-hCG levels exceed the discriminatory zone (1500–3500 mIU/mL) without visualization of an intrauterine gestational sac, EP becomes highly likely.

Serial β-hCG monitoring is also essential for evaluating response to MTX therapy, with a decline of at least 15% between days 4 and 7 indicating successful medical management [34]. A transient rise or plateau in β-hCG levels during the initial days after MTX administration is a well-recognized phenomenon and should not be interpreted as treatment failure. Accordingly, current protocols emphasize the percentage change in β-hCG levels between days 4 and 7, with a decline of at least 15% indicating therapeutic response [18,34]. The day 4 peak observed in our cohort highlights the need for cautious interpretation of early β-hCG dynamics to avoid premature escalation of treatment, a pattern also noted in MTX-treated cases.

In conclusion, abnormal β-hCG dynamics constitute a valuable marker for EP suspicion, but are not sufficient as a standalone diagnostic tool. They must always be interpreted in conjunction with clinical and imaging findings.

Overall, our findings are consistent with previously reported data and emphasize that β-hCG trends, while valuable, should not be used as standalone diagnostic criteria. Optimal management of ectopic pregnancy requires integration of clinical presentation, biochemical evolution, imaging findings, and, when available, histopathological confirmation—an approach that is particularly relevant for rare ectopic pregnancy subtypes, where large comparative studies are limited.

In our clinical setting, MTX was reserved for hemodynamically stable patients with early, unruptured T-EP, low or moderate β-hCG levels, and the ability to comply with close follow-up. This approach reflects both local practice patterns and the aim of preserving tubal integrity, particularly in women desiring future fertility or with limited access to minimally invasive surgery. This approach is consistent with international guidelines and previously published data supporting MTX as a safe option in selected cases of unruptured T-EP [18].

### 4.2. Discussion on the Ultrasonographic Features in Patients with EP

TVUS is the imaging method of choice for the diagnosis of EP, allowing precise localization of the gestational sac and differentiation between intrauterine pregnancy, EP, and pregnancy of unknown location. Each type of EP presents specific ultrasonographic characteristics that guide both diagnosis and therapeutic management.

T-EP typically appears as an adnexal mass separate from the ovary, sometimes associated with a tubal ring, yolk sac, or embryonic cardiac activity, while the presence of hemoperitoneum suggests rupture [35]. O-EP presents as a hyperechoic lesion within the ovarian parenchyma and may mimic hemorrhagic cysts, requiring careful anatomical correlation [36]. In I-EP is characterized by an eccentrically located gestational sac with a thin surrounding myometrial mantle and the interstitial line sign [22]. C-EP is identified as a gestational sac located within the cervical canal, below the internal os, with or without embryonic activity, and showing intense peritrophoblastic blood flow at Doppler (“flame sign”). As in other studies and our own cases, the endometrium was empty, and the cervix was dilated, with deep implantation of the conceptus [24,34]. CSP was visualized as a gestational sac located in the lower uterine segment, embedded in the anterior uterine wall, above or at the isthmic level, within a CS scar defect. In our cases as well, the anterior myometrial layer was markedly thinned (<3 mm) between the sac and the bladder. Doppler imaging demonstrated increased vascularity surrounding the gestational sac [37]. Differential diagnosis with an ongoing miscarriage or C-EP was essential and was based on the exact position of the sac relative to the endocervical canal.

In all cases, early TVUS—ideally combined with dynamic β-hCG monitoring—allowed for rapid diagnosis and prevention of severe complications. In our cohort, subtype-specific ultrasonographic characteristics showed consistent correlation with biochemical evolution and histopathological confirmation, reinforcing their clinical relevance and diagnostic reliability in guiding individualized management strategies.

### 4.3. Discussion on the Macroscopic and Microscopic Histopathological Findings in Patients Diagnosed with EP

A key strength of this study is the integrated interpretation of clinical presentation, biochemical evolution, TVUS features, and histopathological findings. Rather than being analyzed in isolation, these components were correlated for each EP subtype, allowing a comprehensive understanding of disease behavior, therapeutic response, and underlying tissue alterations.

The histopathological examination of tissue obtained through surgery or curettage remains the definitive methos for confirming the diagnosis of EP and determining the exact site of chorionic villi and trophoblastic implantation. Although clinical and imaging assessments are essential for the initial diagnosis, the anatomopathological evaluation provides diagnostic certainty and excludes critical differential diagnoses such as incomplete miscarriage or trophoblastic tumors.

T-EP is macroscopically characterized by a fallopian tube enlarged in volume, congested, often with a thinned wall and evident rupture, accompanied by intraperitoneal hemorrhage. Microscopically, immature or degenerative chorionic villi infiltrating the tubal mucosa or muscular wall are observed, accompanied by trophoblastic cells, hemorrhage and necrosis [14,19].

O-EP, which may macroscopically mimic a hemorrhagic cyst, required histological confirmation, revealing chorionic villi implanted within the ovarian parenchyma in accordance with Spiegelberg’s criteria [20,38].

I-EP occurs within the myometrial thickness at the interstitial portion of the fallopian tube. Histologically, chorionic villi and trophoblast are deeply implanted in the myometrium, accompanied by hemorrhage and, in some cases, focal rupture [22].

C-EP is a rare variant, most frequently diagnosed through curettage with histopathological identification of chorionic villi and trophoblastic tissue within the cervical stroma, without normal endometrial tissue, and are accompanied by deep hemorrhage and necrosis, but without myometrial invasion [24].

CSP is typically diagnosed by imaging, yet histological confirmation remains crucial. Macroscopically, we observed a gestational sac implanted within the fibrotic lower uterine segment, often associated with marked thinning of the uterine wall. Microscopically, chorionic villi were implanted within dense fibrous and scarred myometrial tissue, without communication with the endometrial cavity. Surrounding the villi, we noted degenerative trophoblast, hemorrhage, and a local inflammatory response, with potential for deep invasion—consistent with previous reports in the literature [26].

Beyond its diagnostic role, histopathological examination provides important clinical insight into the biological response to MTX therapy. In cases managed conservatively, particularly CS pregnancies treated with local MTX injection, the identification of degenerative chorionic villi, trophoblastic apoptosis, fibrinoid necrosis, and reduced villous vascularization reflects effective cytotoxic action at the tissue level. These findings reinforce the complementary role of histopathology in post-treatment assessment, especially in rare ectopic pregnancy locations where clinical and imaging data alone may be insufficient [37,39].

Overall, clinical–imaging–histopathological correlation remains particularly relevant in uncommon ectopic pregnancy subtypes, contributing to accurate diagnosis, informed therapeutic decision-making, and improved understanding of treatment response.

### 4.4. Study Limitations

This study has several limitations that should be acknowledged. Its retrospective and single-center design limits the generalizability of the findings and introduces potential selection bias. Therefore, the results should be interpreted with caution and may not be directly generalizable to broader or population-based settings. In addition, the relatively small sample size—particularly for rare EP subtypes—restricts statistical power and precludes definitive conclusions; therefore, findings related to these rare subtypes should be regarded as descriptive and hypothesis-generating rather than confirmatory.

However, these limitations largely reflect the intrinsic rarity of certain EP locations rather than methodological shortcomings. Despite these constraints, the study provides a comprehensive clinical-imaging and histopathological correlation within a well-characterized cohort, offering valuable descriptive and educational insights. This integrative approach may support clinical decision-making in complex or uncommon EP presentations and serve as a foundation for future multicenter or prospective studies.

Additionally, clinical symptoms at presentation were not systematically recorded in all cases, limiting a quantitative assessment of the classical EP triad.

## 5. Conclusions

EP remains a complex pathology that is strongly influenced by patient profiles and modern epidemiological patterns, including advanced reproductive age, obstetrical history, and the increasing use of ART. Accurate diagnosis requires the rigorous integration of clinical findings, β-hCG dynamics, and high-resolution imaging. TVUS remains the key tool for the early identification of T-EP, O-EP, I-EP, C-EP, and CSP, each characterized by distinct morphological and vascular features, as reflected both macroscopically and microscopically.

In rare forms, characterized by deep invasion and major hemorrhagic risk, prompt recognition of subtle imaging signs and their correlation with the biological context represent critical steps in preventing complications. However, histopathological examination remains the only definitive method, demonstrating the presence of ectopically implanted chorionic villi and trophoblast, together with characteristic hemorrhagic and necrotic changes.

Overall, the management of EP requires a multimodal approach that optimizes collaboration among clinicians, imagers, and pathologists, thereby improving diagnostic accuracy and patient safety and reducing morbidity associated with this potentially severe entity. Although limited by its retrospective design and sample size, particularly for rare EP subtypes, this study highlights the practical value of a comprehensive, multidisciplinary diagnostic approach and may serve as a reference framework for the evaluation and management of complex or uncommon EP presentations.

## Data Availability

All data presented here are available from the authors upon reasonable request.

## Supplementary Materials

The following supporting information can be downloaded at https://www.mdpi.com/article/10.3390/jcm15020507/s1, Table S1: Demographic and clinical characteristics of patients included in the study, according to ectopic pregnancy subtype.

## Abbreviations

The following abbreviations are used in this manuscript:

APC	Argon Plasma Coagulation
ARTs	Assisted Reproductive Technologies
C-EP	Cervical Ectopic Pregnancy
Co-EP	Cornual Ectopic Pregnancy (gestational sac implanted at the junction between the uterine cavity and the interstitial portion of the tube)
CRL	Crown–Rump Length
CS	Cesarean Section
CSP	Cesarean Scar Pregnancy
EP	Ectopic Pregnancy
ESI	Extensive Surgical Intervention
Ex-EP	Extrauterine Ectopic Pregnancy
FIV/IVF	In Vitro Fertilization
HE	Hematoxylin–Eosin Staining
HP	Heterotopic Pregnancy (coexistence of intrauterine and extrauterine pregnancy)
I-EP	Interstitial Ectopic Pregnancy
MTX	Methotrexate Medical Treatment
NE	Normal Delivery (equivalent to VD)
NP	Normal Progression (toward the uterine cavity)
O-EP	Ovarian Ectopic Pregnancy
PAS	placenta accreta spectrum
R	Rural Area
SE	Spontaneous Evacuation
SUE	Surgical Uterine Evacuation
T-EP	Tubal Ectopic Pregnancy
TVUS	Transvaginal Ultrasound
TAUS	transabdominal ultrasonography
U	Urban Area
U-EP	Uterine Ectopic Pregnancy
UPO	Unilateral Partial Oophorectomy
US	Unilateral Salpingectomy
US-l	Unilateral Laparoscopic Salpingectomy
US-L	Unilateral Salpingectomy via Laparotomy
VD	Vaginal Delivery
WHO	World Health Organization
β-hCG/hCG	Beta-Human Chorionic Gonadotropin
