# Supplementary material for: From Suspicion to Confirmation: An Original Study on a Complete Diagnostic Pathway for Ectopic Pregnancy
Source: J Clin Med. 2026 Jan 8;15(2):507. doi: 10.3390/jcm15020507 (PMC12842302; doi:10.3390/jcm15020507)
Supplement: Supplementary file 1 [file jcm-15-00507-s001.zip › jcm-4035985-supplementary.pdf]

Supplementary Table S1

Demographic and clinical characteristics of patients included in the study, according to ectopic pregnancy subtype

EP subtype	N	Mean age ± SD (years)	Urban (%)	Rural (%)	Prior CS (%)	Prior VD (%)	Prior abortions (%)	Prior gynecologic surgery (%)
**T-EP – US-l**	10	30.71 ± 2.51	70	30	40	30	50	40
**T-EP – US-L**	14	33.29 ± 4.34	64.29	35.71	35.71	28.57	35.71	42.86
**T-EP - MTX**	10	27.9 ± 3.81	50	50	0	40	30	0
**T-EP – MTX – US-l**	2	26 ± 2.83	70	30	0	0	0	0
**Ovarian EP (O-EP)**	2	33.0 ± 4.2	100	0	50	50	0	50
**Heterotopic pregnancy (HP)**	2	36.5 ± 0.7	50	50	100	50	0	100
**Cornual EP (Co-EP)**	2	28.5 ± 3.5	50	50	50	0	0	0
**Interstitial EP (I-EP)**	1	28	100	0	100	0	0	0
**Cervical EP (C-EP)**	5	33.0 ± 3.3	40	60	60	40	60	0
**Cesarean scar pregnancy (CSP)**	12	30.2 ± 3.6	55	45	72.73	54.55	45.45	45
**Total**	**60**	**—**	**62**	**38**	**48**	**42**	**40**	**38**

Abbreviations

EP, ectopic pregnancy; T-EP, tubal ectopic pregnancy; O-EP, ovarian ectopic pregnancy; HP, heterotopic pregnancy; Co-EP, cornual ectopic pregnancy; I-EP, interstitial ectopic pregnancy; C-EP, cervical ectopic pregnancy; CSP, cesarean scar pregnancy; CS, cesarean section; VD, vaginal delivery.
